# High-Temperature Electrical Transport Behavior of p-Doped Boron Diamond Film/n-WS_2_ Nanosheet Heterojunction

**DOI:** 10.3390/nano15241900

**Published:** 2025-12-18

**Authors:** Changxing Li, Dandan Sang, Yarong Shi, Shunhao Ge, Lena Du, Qinglin Wang

**Affiliations:** 1 School of Physics Science and Information Technology, Key Laboratory of Quantum Materials Under Extreme Conditions in Shandong Province, Liaocheng University, Liaocheng 252000, China; 2 Department of Physics, Capital Normal University, Beijing 100048, China

**Keywords:** WS_2_ nanosheets, B-doped diamond, surface termination, high temperature

## Abstract

WS_2_ is a promising material for applications in wearable devices, field-effect transistors, and high-performance heterojunctions. However, significant challenges remain regarding effective regulation and temperature stability. This study investigates the temperature-dependent electrical properties of WS_2_ heterojunctions prepared by electrophoretic deposition on boron-doped diamond films. The results reveal that the rectification ratio of lightly doped boron heterojunctions at room temperature is 9.1, indicating thermal excitation behavior at temperatures above 100 °C. In contrast, heavily doped boron heterojunctions maintain a rectification ratio consistently below 1 over a temperature range from room temperature to 180 °C, indicating reverse rectification. The lowest rectification ratio observed at 140 °C is 0.17. Density functional theory (DFT) calculations suggest that hydrogen (H) termination generates an internal electric field in the opposite direction, causing a reversal of the rectification polarity, while oxygen (O) termination favors forward rectification. Additionally, due to vacancy defects in WS_2_, the heterojunction exhibits negative differential resistance at 120 °C, with a peak-to-valley ratio of 2.4. Higher doping levels, in comparison to lower concentrations, offer a more stable rectification ratio at elevated temperatures, making the material more suitable for high-temperature, high-frequency, and high-power applications.

## 1. Introduction

In the ongoing pursuit of higher efficiency, smaller size, and enhanced flexibility in electronic devices, semiconductor materials with high-temperature stability and tunable performance have become pivotal for the next generation of power electronics, deep space exploration, and extreme environmental sensing. Traditional semiconductors, such as silicon (Si) and gallium arsenide (GaAs), are hindered by narrow bandgaps and poor thermal conductivity, posing significant challenges for high-temperature applications [1].

Wide bandgap semiconductors are widely recognized for their exceptional physical and chemical stability, offering a promising solution to overcome these limitations. Among them, boron-doped diamond (BDD) stands out as a key material for next-generation semiconductor applications due to its ultra-wide bandgap (5.5 eV), the highest thermal conductivity of any known material, excellent chemical inertness, and high breakdown field strength [2]. Numerous diamond-based power devices have already been developed, incorporating materials such as ZnO, TiO_2_, and SiC [3,4,5]. However, the regulation of electronic properties in these heterojunctions is typically reliant on doping methods, and these heterojunctions are not ideal for short-channel or ultra-thin electronic devices, facing considerable limitations in many application scenarios [6].

Two-dimensional transition metal dichalcogenides (TMDCs), particularly tungsten disulfide (WS_2_) nanosheets, offer an ideal platform for constructing van der Waals heterojunctions and enabling tunable band engineering. This is due to their atomic-scale thickness, adjustable bandgap, excellent mechanical flexibility, and unique interlayer transport properties [7]. By combining the extreme environmental resilience of BDD with the tunable electronic properties of WS_2_ nanosheets, it is possible to achieve a performance breakthrough that effectively balances rigidity and flexibility. Similarly, other morphologies (nanotubes) of WS_2_ nanomaterials have also been extensively studied. For example, Zhang et al. [8] reduced the crystal symmetry from simple inversion symmetry to nanotubes with polar properties, greatly enhancing the overall photovoltaic effect. Faella et al. [9] deposited WS_2_ nanotubes onto graphene electrodes, achieving efficient charge transfer. Domain et al. [10] studied the electronic properties of WS_2_ nanotubes under torsional deformation using density functional theory calculations.

In previous experiments, the performance of diamond- and TMDC-based heterojunctions fell short of expectations, with significant changes in their *I-V* characteristics and even rectification reversal occurring over the temperature range from room temperature to 180 °C. However, the underlying mechanisms driving these differences have yet to be fully explored. Additionally, the impact of surface passivation at elevated temperatures on electronic transport characteristics has not been thoroughly demonstrated [11,12,13].

In this paper, we investigate the performance evolution of a heterojunction composed of BDD thin films and WS_2_ nanosheets under high-temperature conditions. Through a combination of temperature-dependent electrical characterization and theoretical simulations, we reveal the carrier transport mechanism of the heterojunction at elevated temperatures. The results demonstrate that the heterojunction with light boron doping consistently exhibits forward rectification, with a rectification ratio of 9.1 at room temperature. At temperatures above 100 °C, charge carriers can freely pass through the interface barrier. In contrast, the heterojunction with heavy boron doping maintains a rectification ratio below 1 across the temperature range from room temperature to 180 °C, displaying reverse rectification characteristics. The rectification ratio reaches its minimum value of 0.17 at 140 °C. Moreover, at 120 °C, this heterojunction exhibits negative differential resistance, with a peak-to-valley ratio of 2.4.

Density functional theory (DFT) calculations indicate that hydrogen (H) capping increases the work function, which in turn induces an opposite built-in electric field, providing insight into the intrinsic mechanism of current reversal. Additionally, sulfur (S) vacancy defects in WS_2_ generate new defect energy levels within the forbidden band, facilitating interband tunneling at 120 °C and resulting in a negative impedance effect. This study provides a critical material platform and design foundation for the development of next-generation, high-performance, extreme environment-resistant, and functionally reconfigurable electronic devices.

## 2. Materials and Methods

WS_2_ nanosheets were prepared using the liquid-phase exfoliation method. First, WS_2_ powder (Macklin, Shanghai, China) was immersed in deionized water for 1 h. Next, 120 mg of dried WS_2_ powder was dispersed into a mixed solution of 40 mL isopropanol (Macklin, Shanghai, China) and water (in a 3:7 ratio). The mixture was subjected to ultrasonic treatment for 4 h, followed by centrifugation at 3000 rpm for 15 min to collect the upper suspension. The ultrasonic treatment was repeated three times to obtain a stable colloidal suspension.

Thin films of lightly doped boron diamond (LBDD) and heavily doped boron diamond (DBDD) were fabricated using hot wire chemical vapor deposition (HWCVD). The heat source was provided by a metal wire, with trimethyl borate and methane serving as the boron and carbon sources, respectively. The working pressure was maintained at 5–8 kPa, and the deposition process lasted for 4.5 h. During this time, the working temperature was kept at 700–900 °C.

The P-LBDD film/n-WS_2_ nanosheet and p-DBDD film/n-WS_2_ nanosheet heterojunctions were fabricated using the electrophoretic deposition method. The diamond film was placed on the positive electrode of the power supply, while a platinum plate was used as the negative electrode. A parallel electric field was established, and the assembly was immersed in a colloidal suspension. The deposition was carried out under a voltage of 30 V for a duration of 2 h. After the deposition, the samples were removed and allowed to dry naturally.

DFT calculations were performed using the DMOL3 code (Materials Studio 2020, Dassault Systèmes, Paris, France), with the Perdew-Burke-Ernzerhof (PBE) exchange-correlation functional in the Generalized Gradient Approximation (GGA). All calculations were conducted with DNP basis sets. A vacuum layer of 20 Å was applied along the non-periodic direction to minimize periodic mirror interactions. The electronic relaxation threshold was set to 10^−6^ eV, while the energy threshold for ion relaxation was set to 10^−5^ Ha, and the force threshold was set to 0.002 Ha/Å. For the WS_2_ system, a 3 × 3 × 1 supercell was used, and for the diamond system, a 2 × 2 × 1 supercell was employed to ensure the lattice mismatch of the heterojunction remained below 5%. The Monkhorst–Pack grid was set to 3 × 3 × 1 for both systems.

The morphology and structure of the samples were examined using scanning electron microscopy (SEM, Thermo Fisher Scientific FIB-SEM GX4, Waltham, MA, USA) and atomic force microscope (AFM, Bruker, Billerica, MA, USA). The elemental composition of the samples was analyzed by energy-dispersive X-ray spectroscopy (EDX, Thermo Fisher Scientific FIB-SEM GX4, America). The phase structure and phase purity of the samples were examined using X-ray diffractometry with Cu Kα radiation (XRD, Rigaku SmartLab, Tokyo, Japan). The *I-V* characteristics of the heterojunction were measured using a Keithley 2400 source (Keithley Instrument, Cleveland, OH, USA). Raman measurement (InVia Reflex, Renishaw, UK) was made using a 532 nm laser for excitation. Absorption of the materials was obtained by a home-built setup using an optical spectrometer and an objective lens.

## 3. Results and Discussion

**Figure 1:**
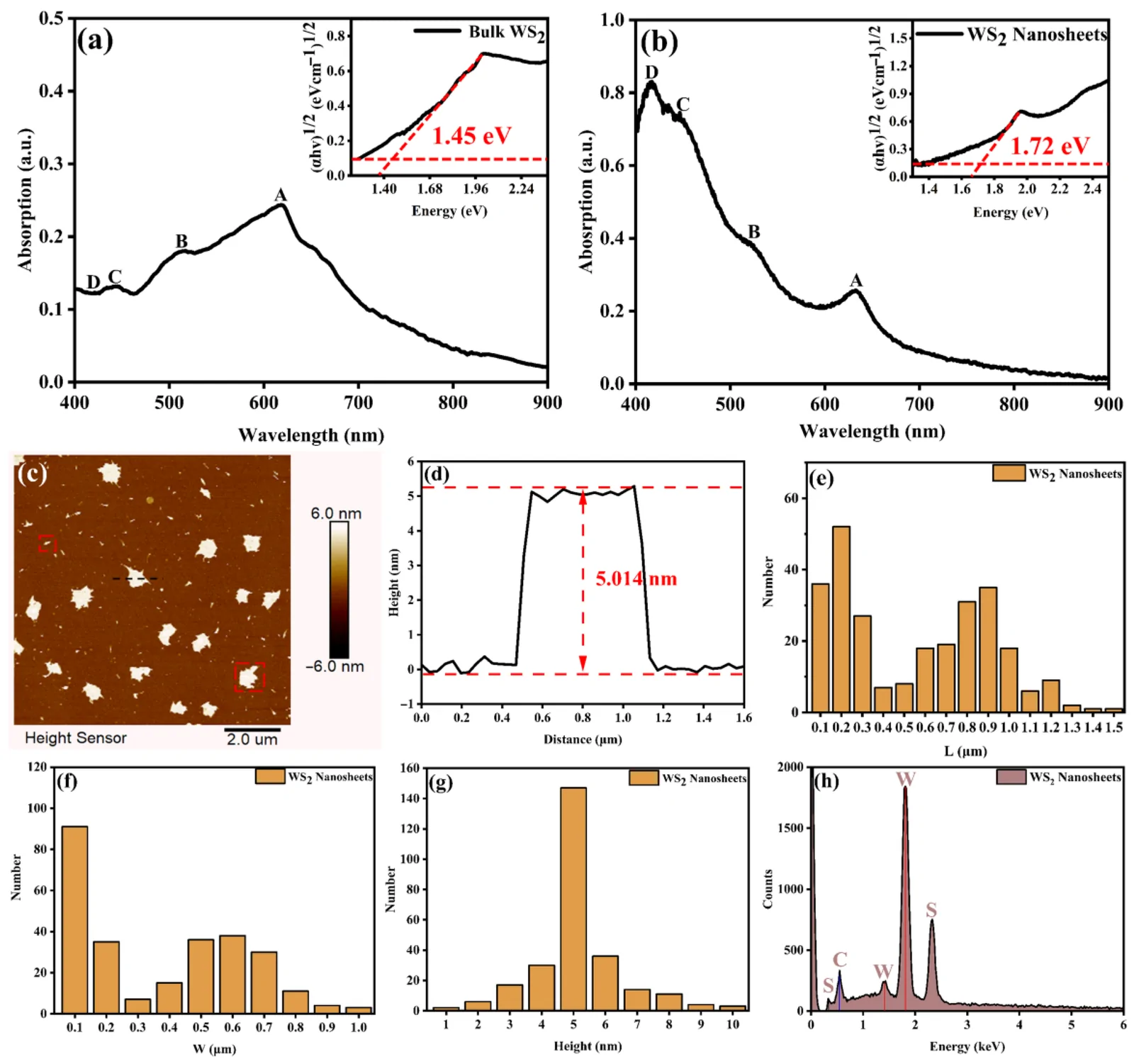
(**a**) Absorption spectrum of bulk WS_2_ (illustration shows the optical bandgap of bulk WS_2_). (**b**) The absorption spectrum of WS_2_ nanosheets (the illustration shows the optical bandgap of WS_2_ nanosheets). (**c**,**d**) AFM images of WS_2_ nanosheets. (**e**–**g**) Statistical measurement of 200 WS_2_ nanosheets at different locations. (**h**) EDX of WS_2_ nanosheets.

The absorption spectra of WS_2_ nanosheets and the original bulk material are presented in Figure 1a,b. In the bulk material, two prominent absorption peaks are observed at 618 nm and 517 nm, corresponding to the A and B exciton characteristics, respectively. These exciton features arise from the splitting of the valence band due to spin–orbit coupling at the K point in the Brillouin zone [14,15]. In contrast, for the dispersed WS_2_ nanosheets, the A and B exciton peaks undergo a redshift, appearing at 630 nm and 519 nm, respectively. Additionally, two additional high-resolution absorption peaks are observed at 446 nm and 416 nm, corresponding to the C and D excitons, respectively. These C and D excitons emerge due to band renormalization effects induced by interlayer coupling, and they are only observed in few-layer WS_2_. The C exciton originates from the nested region of the conduction and valence bands, displaying strong light absorption due to the van Hove singularity [16]. The D exciton, on the other hand, results from a transition between the valence band peak and the conduction band valley at the Γ point in the Brillouin zone [17].

To further investigate the evolution of the optical bandgap, we performed calculations and coordinate transformations. The initial optical bandgap for the bulk WS_2_ material is 1.45 eV, whereas for the WS_2_ nanosheet sample, the optical bandgap is 1.72 eV. This bandgap widening is consistent with the layer-dependent variation in bandgap (1.3–2.1 eV), highlighting the significant influence of layer reduction on the material’s optical properties.

To comprehensively characterize the morphology and thickness of WS_2_ nanosheets, AFM measurements were performed on samples uniformly dispersed on a mica substrate, as shown in Figure 1c–g. A total of 200 nanosheets, randomly selected from various positions, were analyzed. The nanosheets exhibited irregular shapes and a distinct bimodal size distribution. Notably, the thickness distribution was highly uniform, with an average thickness of approximately 5 nm, corresponding to around 6 to 10 layers [18].

The EDX of WS_2_ was measured by assembling WS_2_ nanosheets on a diamond film substrate using electrophoretic deposition, as shown in Figure 1h. EDX analysis confirmed the predominant presence of tungsten (W) and S elements, with normalized mass fractions of 76.82% and 23.18%, respectively. The atomic ratio of W to S is about 1:1.73, deviating from the ideal stoichiometric ratio of 1:2. Therefore, it can be qualitatively assumed that there are S vacancies in WS_2_ [19]. There is a C element signal of the diamond substrate in the EDX spectrum. Compared with W and S, the peak of element C is very weak. In addition, no other impurity elements were observed.

**Figure 2:**
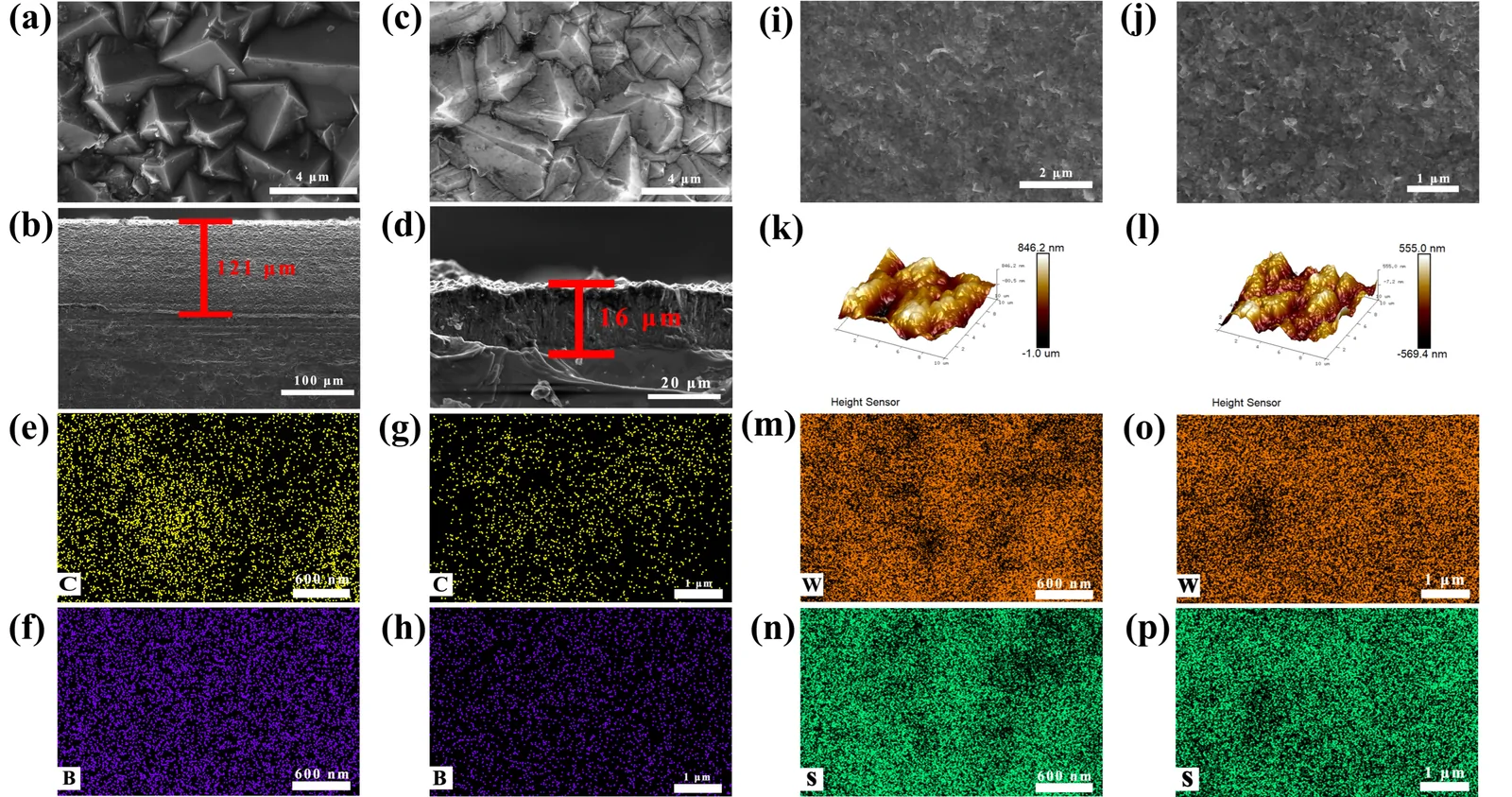
(**a**) SEM of the lightly doped boron diamond thin film. (**b**) Cross-section SEM of the lightly doped boron diamond thin film. (**c**) SEM of the heavily doped boron diamond thin film. (**d**) Cross-section SEM of the heavily doped boron diamond thin film. (**e**,**f**) Element surface distribution of the lightly doped boron diamond thin film. (**g**,**h**) Element surface distribution of the heavily doped boron diamond thin film. (**i**) SEM of the lightly doped heterojunction. (**j**) SEM of the heavily doped heterojunction. (**k**) AFM of the lightly doped heterojunction. (**l**) AFM of the heavily doped heterojunction. (**m**,**n**) Element surface distribution of the lightly doped heterojunction. (**o**,**p**) Element surface distribution of the heavily doped heterojunction.

Figure 2a–d present the surface morphologies of lightly boron-doped diamond (LBDD) and heavily boron-doped diamond (HBDD) films grown on silicon substrates. Both films are composed of densely packed, pyramidal diamond grains with sizes ranging from 1 μm to 3 μm. Distinct morphological differences are evident between the two samples, most notably the more pronounced twinning observed in the HBDD film. The formation of twins is attributed to secondary nucleation processes induced by boron impurities during film growth [20]. The LBDD film exhibits a thickness of approximately 121 μm, whereas the HBDD film is about 16 μm thick.

Elemental spatial mapping, shown in Figure 2e–h, further reveals the distribution of carbon and boron in the two thin films. Given the significant influence of doping on carrier transport behavior, Hall effect measurements were performed. The LBDD film exhibits a carrier concentration of 2.3 × 10^17^ cm^−3^, a resistivity of 32.2 Ω·cm, and a mobility of 27.5 cm^2^·V^−1^·s^−1^. In contrast, the HBDD film shows a markedly higher carrier concentration of 3.9 × 10^20^ cm^−3^, accompanied by a mobility of 11.3 cm^2^·V^−1^·s^−1^ and a resistivity of 12.5 Ω·cm.

The SEM images depicting the assembly of WS_2_ nanosheets on the surface of BDD are shown in Figure 2i,j. In both heterojunctions, the diamond particles are entirely covered by the nanosheets. These nanosheets are arranged parallel to the substrate in a staggered manner, forming a film structure [21]. AFM measurements (Figure 2k,l) further characterize the surface morphology. The root mean square (RMS) roughness of the nanosheet thin film in the lightly doped heterojunction (LDH) is approximately 233 nm, whereas that of the heavily doped heterojunction (HDH) decreases to 139 nm. The reduced surface roughness indicates a smoother and more homogeneous film surface, which is essential for achieving optimal electrode contact. It should be noted that the thickness in Figure 1c–g refers to the thickness of a single WS_2_ nanosheet. After electrophoretic deposition, multiple nanosheets are stacked together to form a thin film morphology, resulting in greater roughness. In addition, due to the roughness of the diamond substrate, we cannot obtain the exact thickness of the WS_2_ film, but the thickness of the WS_2_ film is roughly a few micrometers. Additionally, the size of diamond particles is about 1–3 μm, while the roughness of heterojunctions is on the nanometer scale, indicating that the thickness of WS_2_ thin films is uneven, which may disrupt the ideal parallel plate model, induce uneven depletion region width and tunneling probability differences, leading to additional carrier recombination and tunneling behavior. A roughness greater than 100 nm indicates a slight height difference on the surface of the heterojunction. This may result in a slightly smaller effective contact area than the surface area of the heterojunction. Therefore, it will lead to a decrease in the experimental value of the current. Furthermore, elemental surface distribution analysis (Figure 2m–p) displays that the distribution of W and S on the film, indicating that the film has continuity and no obvious pores.

**Figure 3:**
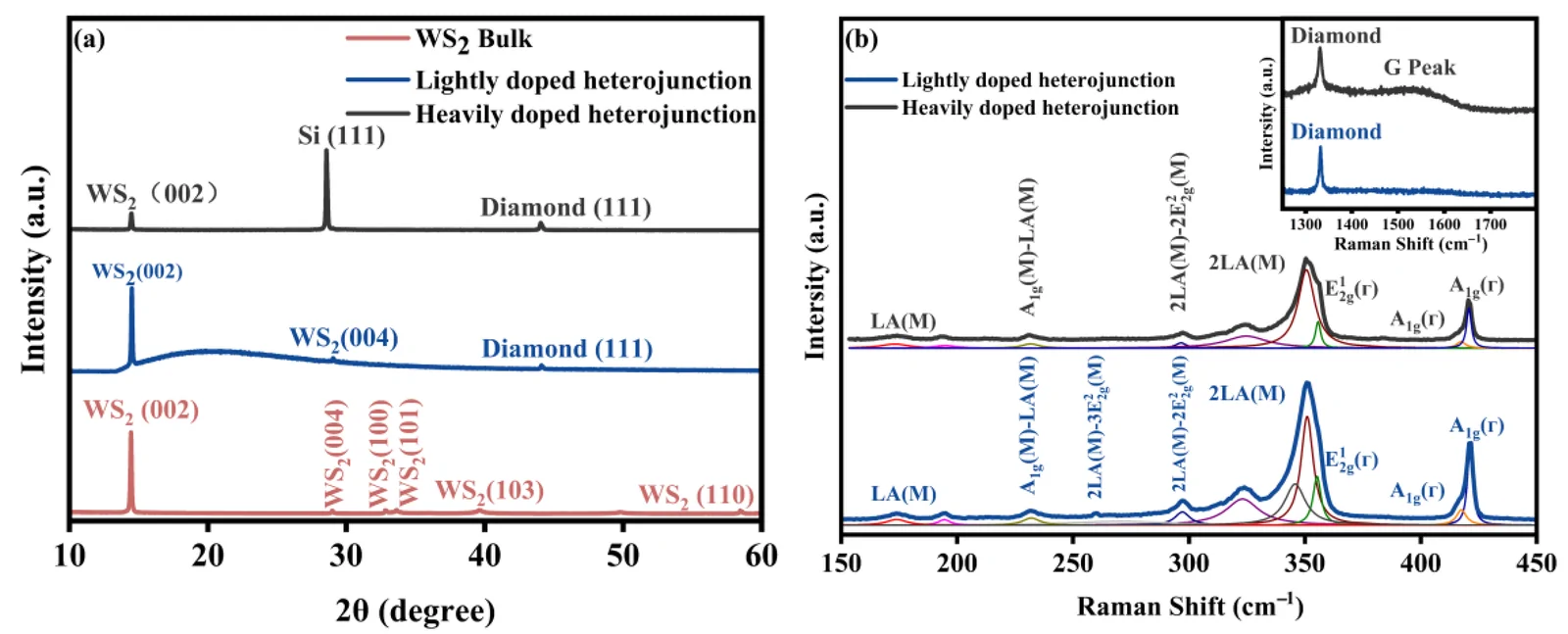
(**a**) XRD diagram of bulk WS_2_, lightly doped heterojunction, and heavily doped heterojunction. (**b**) Raman spectra of a lightly doped heterojunction and a heavily doped heterojunction. The colored lines represent the fitting results.

The crystal structures of the LDH, HDH, and bulk WS_2_ samples were analyzed using XRD, as presented in Figure 3a. The pristine bulk WS_2_ sample exhibited multiple distinct diffraction peaks, consistent with the standard 2H crystal structure (JCPDS card No. 08-0237) [22]. In the XRD pattern of the LDH sample, characteristic diffraction peaks at 14.22° and 28.98° were identified, corresponding to the (002) and (004) crystal planes of 2H-WS_2_, respectively. Additionally, a diffraction peak at 44.08° was attributed to the (111) crystal plane of LBDD [23].

Similarly, the HDH sample displayed diffraction peaks at 14.21° and 28.99°, corresponding to the (002) plane of 2H-WS_2_ and the (111) plane of the Si substrate, respectively. The appearance of the Si diffraction signal is likely due to the relatively small thickness of the HBDD film, which allows X-rays to penetrate to the underlying substrate. The diffraction peak at 44.04° originated from the (111) crystal plane of HBDD [24].

Importantly, the pronounced diffraction from the (002) plane observed in both heterojunctions confirms the highly ordered, layered structure of the WS_2_ nanofilms. This also indicates that the WS_2_ retains its intrinsic 2H-phase crystal structure after liquid-phase exfoliation, with no detectable impurity-related diffraction peaks.

Raman spectroscopy analysis of the heterojunctions (Figure 3b) revealed an overlap between the second-order longitudinal acoustic phonon mode, 2LA(M), and the first-order in-plane vibrational mode, E^1^_2g_ (Γ), of the WS_2_ nanosheet films [25]. To accurately distinguish the contributions of these vibration modes, the Lorentz function fitting method was employed. In the LDH sample, the in-plane vibrational mode E^1^_2g_ (Γ) of the S-W-S bond appears at 355.26 cm^−1^, while the out-of-plane vibrational mode A_1g_ (Γ) exhibits bimodal characteristics at 417.4 cm^−1^ and 420.6 cm^−1^, respectively. Similarly, for the HDH sample, the E^1^_2g_ (Γ) and A_1g_ (Γ) modes are located at 355.6 cm^−1^, 417.7 cm^−1^, and 420.8 cm^−1^, respectively.

According to previous studies, as the number of WS_2_ layers increases, the interlayer phonon restoring force strengthens, while the long-range Coulomb interaction weakens due to electrostatic shielding. This leads to a blue shift of the A_1g_ (Γ) mode and a red shift of the E^1^_2g_ (Γ) mode. When the number of layers reaches four, the frequency difference between these two modes typically increases to about 65 cm^−1^ and then stabilizes [26]. In this work, the frequency differences for LDH were 62.14 cm^−1^ and 65.34 cm^−1^, while those for HDH were 62.1 cm^−1^ and 65.2 cm^−1^, respectively. These results are consistent with the AFM measurements, confirming the coexistence of nanosheets with both fewer than four layers and more than four layers within the samples.

The illustration in the upper right corner shows the Raman characteristic peak of boron-doped diamond. LBDD exhibits a characteristic peak at 1332 cm^−1^, while HBDD exhibits a characteristic peak at 1330 cm^−1^ [23,24]. It is worth noting that the G peak at 1540 cm^−1^ in the HBDD spectrum may be due to the disordered accumulation of graphite phase and surface recombination caused by the significant effect of high-concentration boron doping on the crystallization recombination of diamond. In particular, the increase in boron content promotes the formation of boron hydride and consumes hydrogen atoms in the gas phase, resulting in a change in the c:h ratio and a reduction in diamond crystal quality. While the impurity concentration higher than 10^20^ cm^−1^ will significantly introduce stress, resulting in an increase in Sp^2^ content [27].

**Figure 4:**
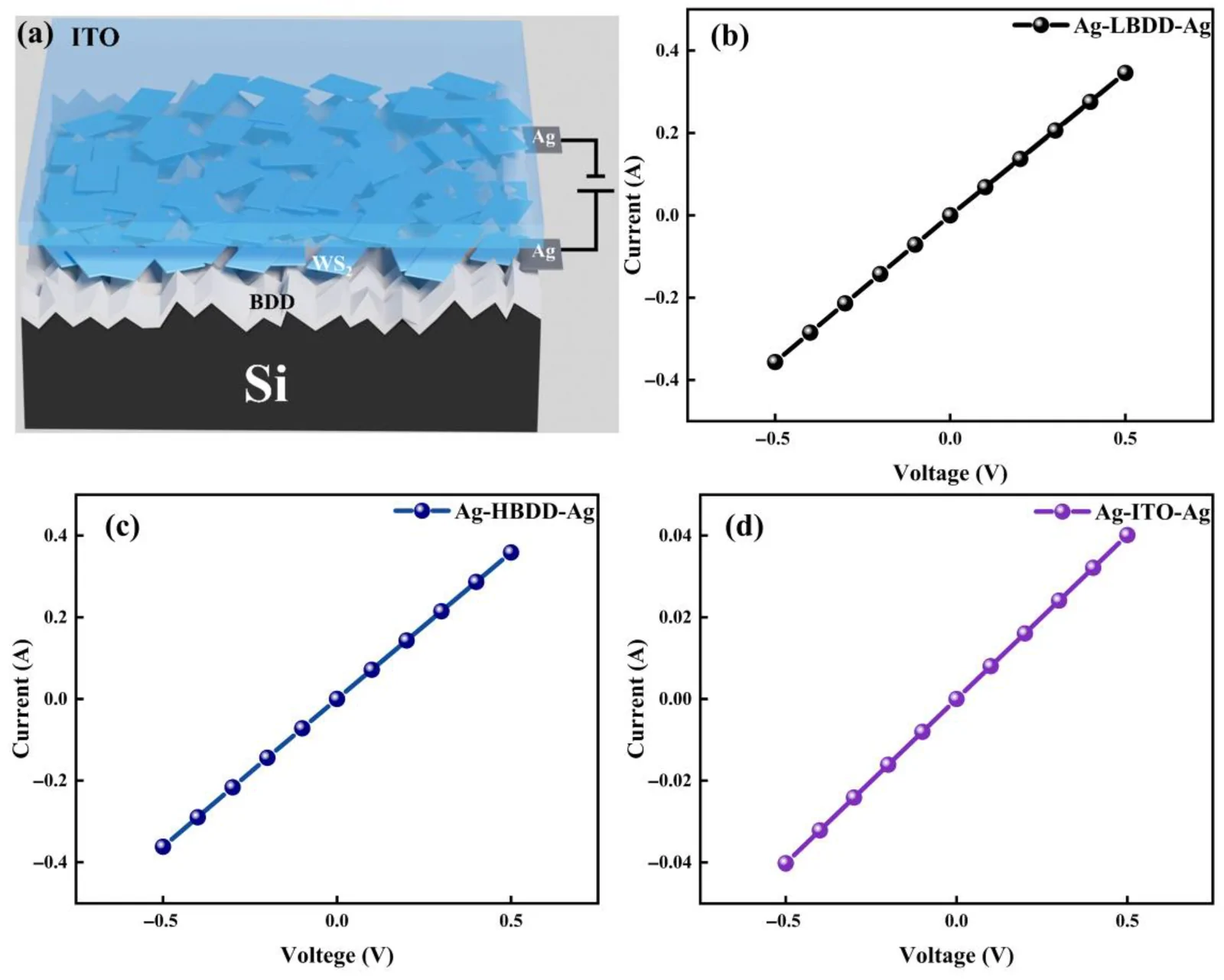
(**a**) Schematic diagram of the device structure. (**b**) *I-V* characteristic curves of Ag-LBDD-Ag. (**c**) *I-V* characteristic curves of Ag-HBDD-Ag. (**d**) *I-V* characteristic curves of Ag-ITO-Ag.

Figure 4a presents the structural model of the fabricated device. The heterojunction cathodes were prepared by attaching the conductive surface of indium tin oxide (ITO) to the WS_2_ nanosheet thin films using cyanoacrylate adhesive. The anode of the heterojunction was formed by fixing a conductive wire onto the BDD surface with silver paste. The *I-V* characteristics of the ITO-Ag and BDD-Ag contacts are shown in Figure 4b–d. The ITO-Ag contact exhibits ideal ohmic behavior.

In the subsequent analysis, the LBDD and HBDD samples are considered to possess oxygen-terminated and hydrogen-terminated surfaces, respectively. The ohmic contact between LBDD and Ag is likely attributed to the effect of boron doping, which modifies the electronic structure at the interface. Meanwhile, the ohmic contact observed between HBDD and Ag arises from the lower work function of HBDD compared to that of Ag, resulting in the formation of a hole anti-blocking layer. Furthermore, since the work function of WS_2_ (4.95 eV) is greater than that of ITO (4.5 eV), it is possible to form a low potential barrier contact at the interface [11].

**Figure 5:**
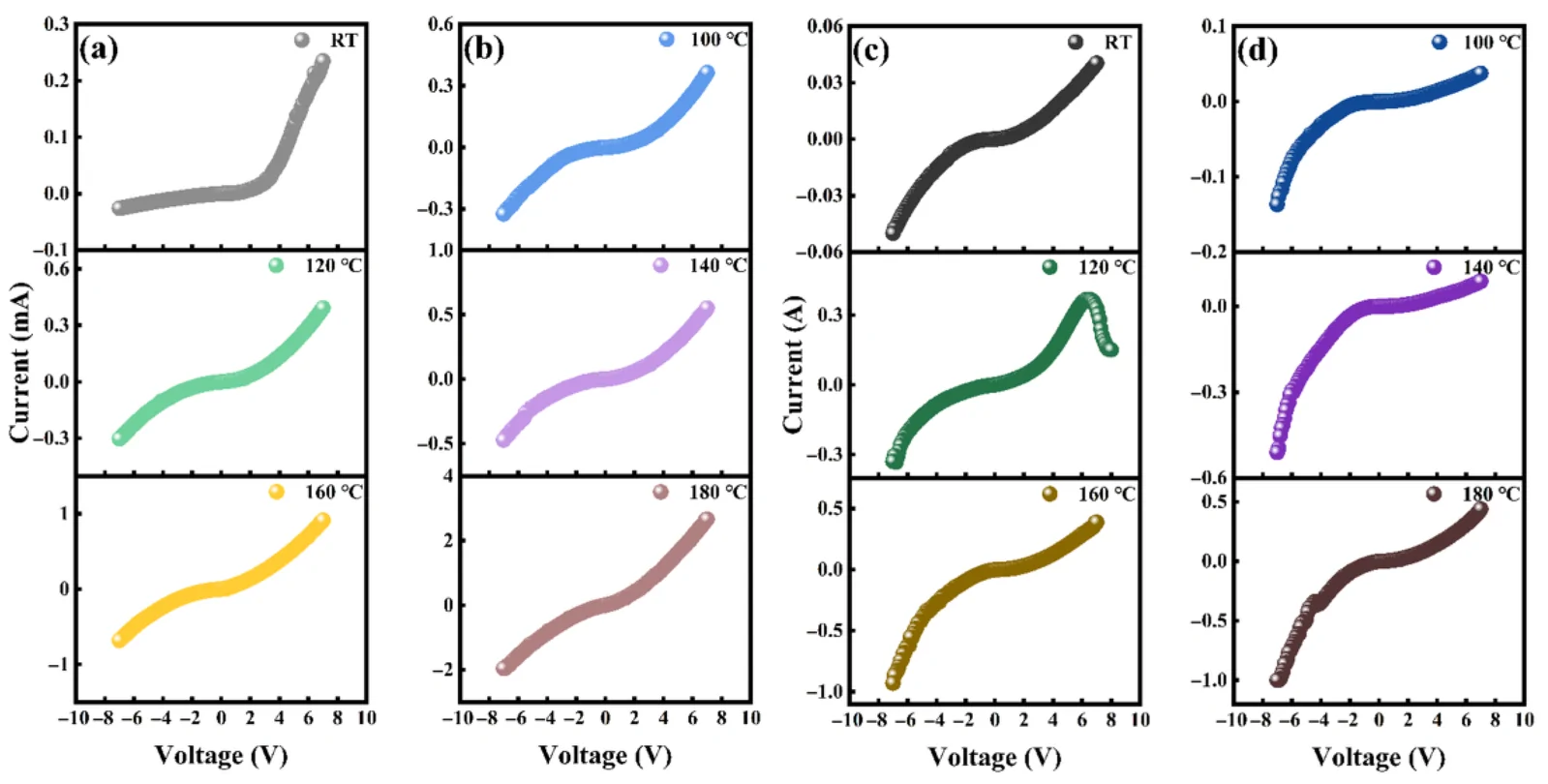
(**a**,**b**) *I-V* characteristic curves of the lightly doped heterojunction at different temperatures. (**c**,**d**) *I-V* characteristic curves of the heavily doped heterojunction at different temperatures.

**Figure 8:**
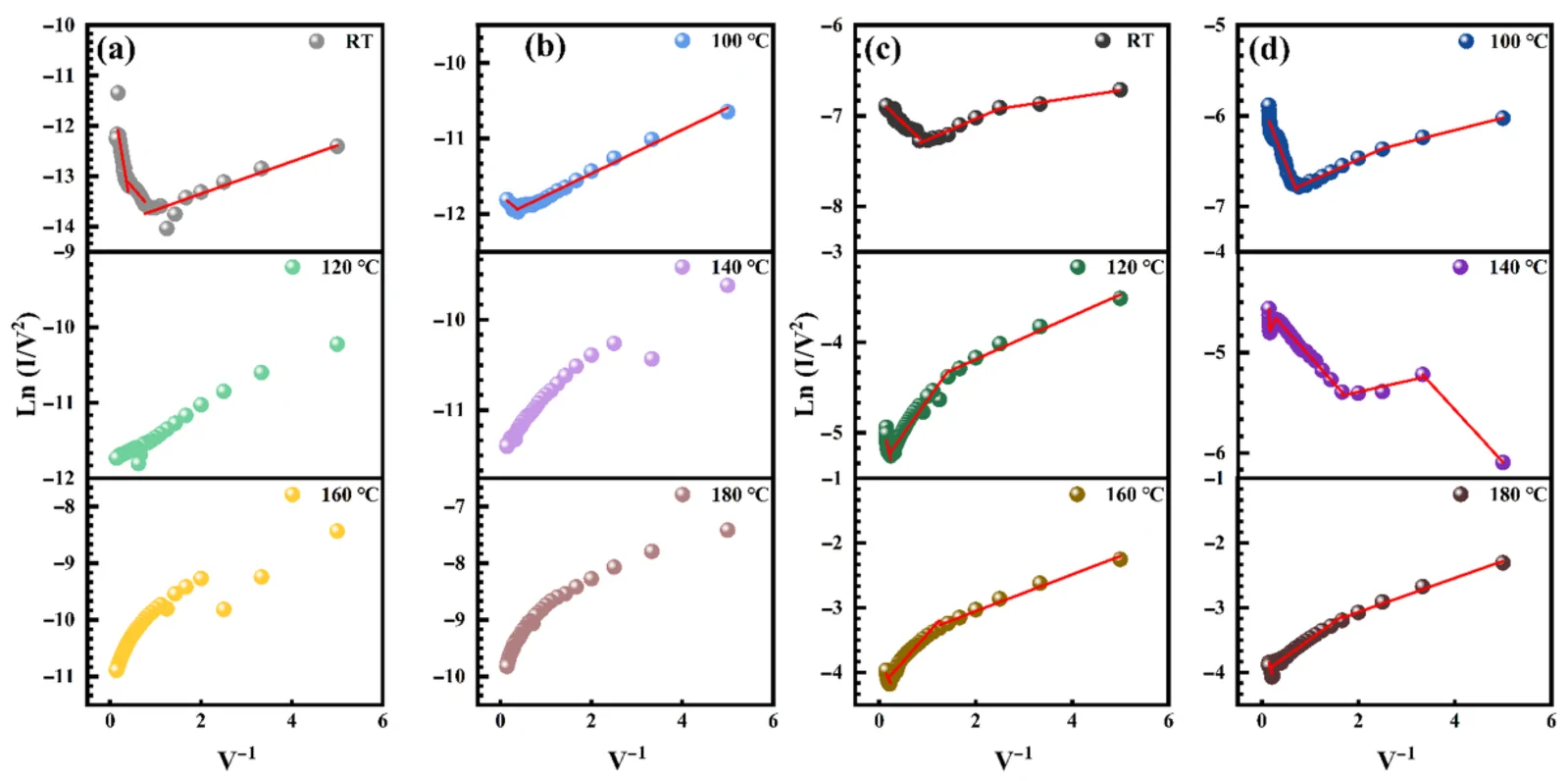
(**a**,**b**) Ln (*I*/*V*^2^) versus 1/*V* diagram of the lightly doped heterojunction at different temperatures. The red line is the fitting result. (**c**,**d**) Ln (*I*/*V*^2^) versus 1/*V* diagram of the heavily doped heterojunction at different temperatures. The red line is the fitting result.

As shown in Figure 5, temperature-dependent electrical measurements were conducted on both LDH and HDH devices. The measurement process is carried out in an air environment, and after equilibration for 10 min at each temperature, actual measurements are taken. The data presented in Table 1 indicate that, at all temperatures, the forward current of the LDH device is consistently greater than the reverse current, with the highest rectification ratio (Defined as the ratio of current between 7 V and −7 V) of 9.1 observed at room temperature. At higher temperatures, the device undergoes thermal excitation, and the rectification ratio approaches 1. This phenomenon can be attributed to carriers acquiring sufficient thermal energy to overcome the potential barrier (Figure 8).

However, this observation contradicts previous studies. The electron affinity of WS_2_ is approximately 3.75 eV, while that of LBDD is around 0.5 eV [28,29]. Due to the low impurity concentration in LDH and the weak n-type behavior of WS_2_, the work function of WS_2_ is approximately 4.61 eV, while that of LBDD is about 3.25 eV. Therefore, at elevated temperatures, the chemical potential causes electrons to diffuse from LBDD into WS_2_, thereby balancing the energy band structure and leading to the formation of a reverse built-in electric field. The most plausible explanation for this phenomenon is the significant change in the work function of diamond, which will be further investigated through DFT calculations.

**Figure 12:**
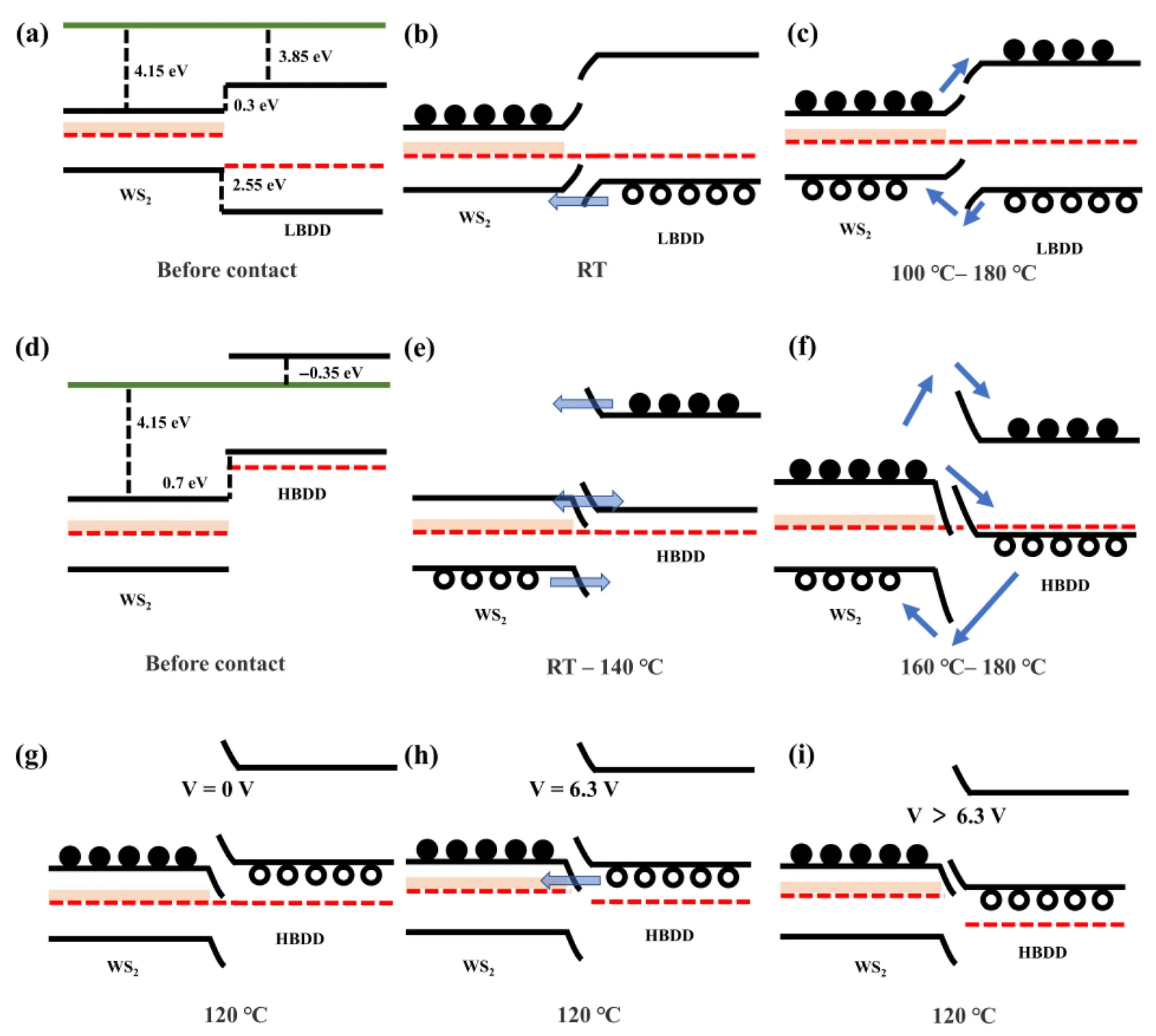
(**a**–**c**) Equilibrium energy band diagram of the lightly doped heterojunction. (**d**–**f**) Equilibrium energy band diagram of the heavily doped heterojunction. (**g**–**i**) Schematic diagram of energy band structure of heavily doped heterojunction at 120 °C under different bias voltages.

For the HDH device, the rectification ratio remains below 1, reaching a minimum value of 0.17 at 140 °C, as shown in Table 2. At 120 °C, a differential negative differential resistance (NDR) phenomenon is observed, with a peak voltage of 6.3 V, a valley voltage of 8.0 V, and a peak-to-valley ratio of 2.4. The origin of the NDR phenomenon may be multifactorial. While in perovskite heterostructures, the negative resistance effect is typically attributed to resonant tunneling; in this case, it is more likely related to defects [30]. Specifically, vacancies in WS_2_ introduce partially occupied defect energy levels within the bandgap, which play a key role in the observed negative resistance behavior. It is worth noting that NDR only exists at a single temperature. As shown in Figure 12, at 120 °C, the defect energy level of WS_2_ aligns with the valence band top of diamond, inducing tunneling. At lower temperatures, the diamond valence band is higher than the defect energy level and therefore cannot effectively induce NDR. At higher temperatures, the valence band of diamond is lower than the defect energy level and is dominated by the behavior of composite charge carriers.

The current of the LDH device is significantly lower than that of the HDH device, which is closely related to the effective contact area and carrier concentration. Additionally, the currents of both samples exhibit typical positive temperature dependence, with an amplitude difference of only one order of magnitude. After excluding data dominated by defects, the relatively stable rectification ratio of HDH suggests that doping enhances the stability of diamond-based heterojunctions. It is worth mentioning that the measured rectification ratio is greater than that of untreated metal oxide p-n diodes (6). The lower rectification ratio is attributed to the presence of some trap state near or at the interface [31].

By combining the semi-logarithmic plot of the *I-V* characteristic curve with the Shakes Miller rectification model, the ideal factors at different temperatures were fitted as follows [32,33]:

(1) $${I=I}_{s}\left[\exp\left(\frac{qV}{nKT}\right)-1\right]$$

Here, *I_s_* is the reverse saturation current, *n* is the ideal factor, *K* is the Boltzmann constant, *T* is the thermodynamic temperature, and *q* is the electron charge.

**Figure 6:**
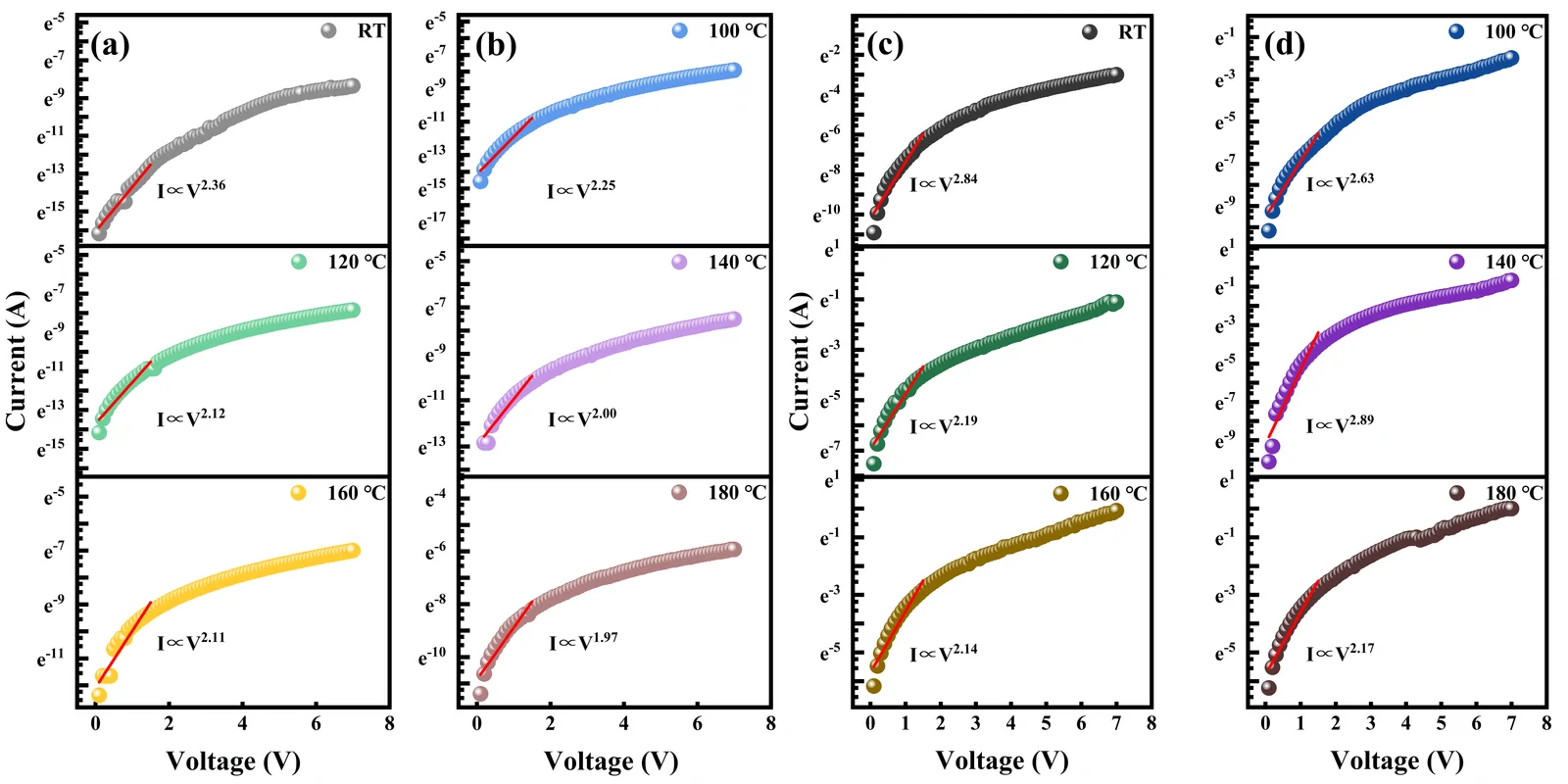
(**a**,**b**) Ln *I*-*V* diagram of the lightly doped heterojunction at different temperatures. The red line is the fitting result. (**c**,**d**) Ln *I-V* diagram of the heavily doped heterojunction at different temperatures. The red line is the fitting result.

In Figure 6, the slope at each temperature is fitted within the range of 0.2–1.5 V, and then the next step is carried out through the differential form of the Schottky diode equation. Starting from 0.2 V is to avoid numerical noise at lower voltages. The ideal factors for LDH are 16.4 at room temperature, 13.8 at 100 °C, 13.9 at 120 °C, 14.0 at 140 °C, 12.7 at 160 °C, and 12.8 at 180 °C. The ideal factors for HDH are 13.5 at room temperature, 11.8 at 100 °C, 13.5 at 120 °C, 7.1 at 140 °C, 12.5 at 160 °C, and 17.7 at 180 °C. At all temperatures, the ideal coefficients of the two heterojunctions are higher than those of the ideal diode (1), but still lower than the results of the prepared MoS_2_/SiNW/n-Si heterojunction (20.0) [34,35]. On the one hand, this is due to lattice mismatch and discontinuity of the band-structure. This can introduce interface states and capture and release charge carriers, increase reverse saturation current, and improve the ideal factor [36]. On the other hand, a larger ideal factor indicates a deviation of the heterojunction from the thermally emission-dominated diffusion current mechanism, suggesting the existence of a recombination-carrier injection mechanism [37]. The slight oscillation of the ideal factor implies that the defect-induced tunneling and carrier injection mechanisms undergo changes under temperature induction. In addition, at higher temperatures, the relative contribution of tunneling current to the total current decreases due to the enhancement of thermal emission current (Figure 8), resulting in a decrease in the ideal factor. The narrow depletion region and degeneracy of HBDD under heavy doping result in a reduction in effective recombination centers within the heterojunction, making its behavior more ideal [38]. Therefore, the ideal factor for HDH is relatively small.

**Figure 7:**
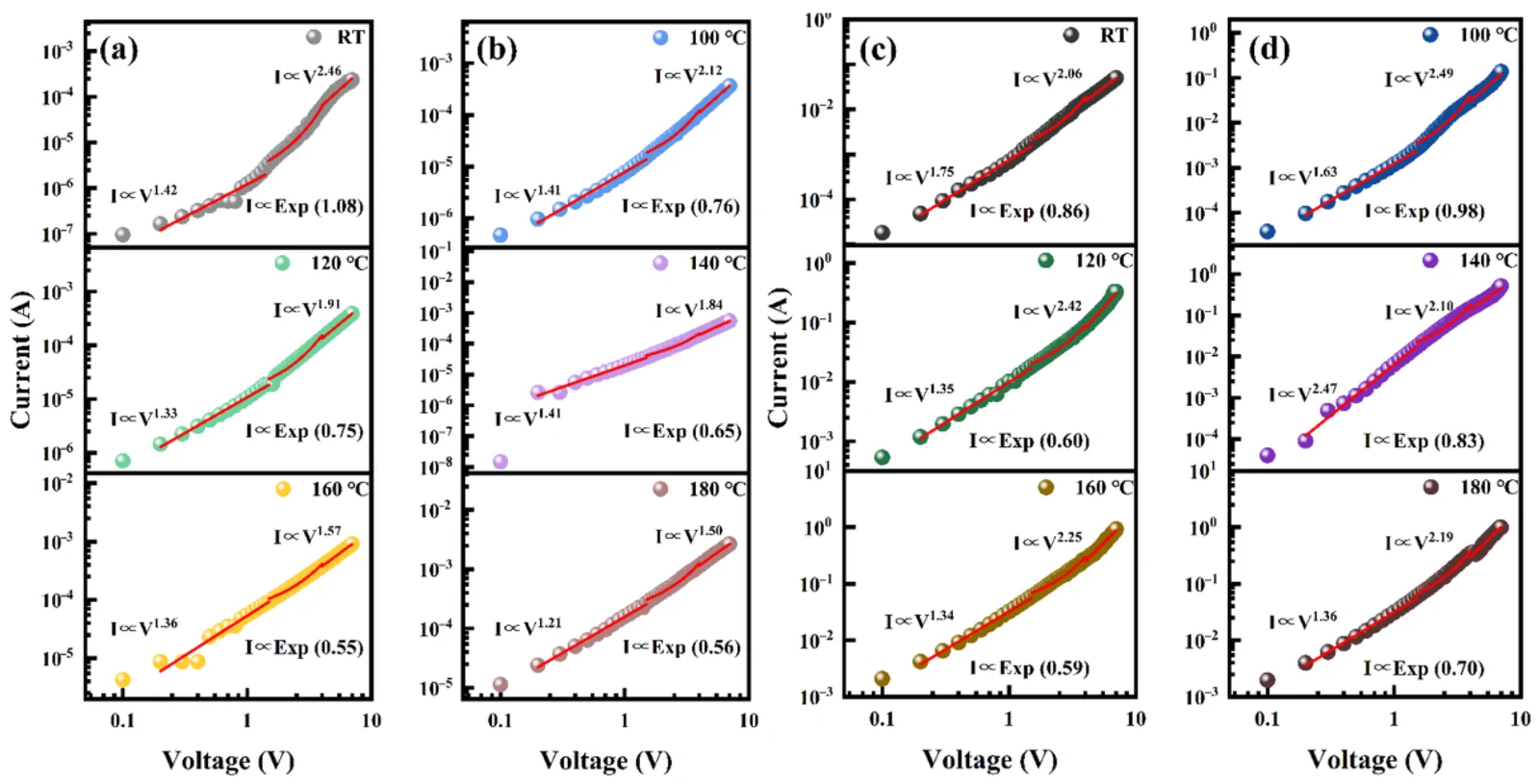
(**a**,**b**) Log *I*-Log *V* diagram of the lightly doped heterojunction at different temperatures. The red line is the fitting result. (**c**,**d**) Log *I*-Log *V* diagram of the heavily doped heterojunction at different temperatures. The red line is the fitting result.

To further investigate the transport mechanisms, the carrier injection of the two heterojunctions at different temperatures was analyzed, as shown in Figure 7. In the low-voltage region, the LDH followed power laws of *I∝V*^1.42^, *I∝V*^1.41^, *I∝V*^1.33^, *I∝V*^1.41^, *I∝V*^1.36^, and *I∝V*^1.21^ at varying temperatures, whereas the HDH followed *I∝V*^1.75^, *I∝V*^1.63^, *I∝V*^1.35^, *I∝V*^2.47^, *I∝V*^1.34^, and *I∝V*^1.36^. An index not greater than 2 indicates that the heterojunction follows Ohmic linearity [39]. The large exponent at 413.15 K in HDH is attributed to the earlier occurrence of Fowler–Nordheim (F-N) tunneling (Figure 8). In the medium-voltage region, the injection efficiencies of the LDH are 1.08 (RT), 0.76 (100 °C), 0.75 (120 °C), 0.65 (140 °C), 0.55 (160 °C), and 0.56 (180 °C), those of the HDH is 0.86 (RT), 0.98 (100 °C), 0.60 (120 °C), 0.83 (140 °C), 0.59 (160 °C), and 0.70 (180 °C). In the high-voltage region, the LDH followed power laws of *I∝V*^2.46^, *I∝V*^2.12^, *I∝V*^1.91^, *I∝V*^1.84^, *I∝V*^1.57^, and *I∝V*^1.50^ at varying temperatures, whereas the HDH followed *I∝V*^2.06^, *I∝V*^2.49^, *I∝V*^2.42^, *I∝V*^2.10^, *I∝V*^2.25^, and *I∝V*^2.19^. Observing an exponent greater than 2 in the high voltage region is consistent with the space charge limited current (SCLC) model [40]. When the density of injected charge carriers is high, these charge carriers accumulate at the interface, and the high-density trap state will capture the charge carriers and generate a built-in electric field opposite to the applied electric field, thereby suppressing subsequent charge carrier injection. According to Mott Gurney’s law, current is proportional to the square of V and inversely proportional to the third power of distance. Exponential behavior with decreasing temperature was observed in LDH, which is related to the decrease in carrier injection efficiency. On the contrary, the irregular changes in carrier injection efficiency and Log *I*-Log *V* index in HDH indicate variations in tunneling dominant carrier injection and strong injected carrier defect interactions.

Figure 8 shows the tunneling mechanism at the heterojunction. Tunneling current is usually modulated by thermal emission, direct tunneling, and F-N tunneling synergy [41]:

Thermionic emission:
(2) $$I=AA^{*}exp[\frac{-(\emptyset_{b}-\sqrt{\frac{q^{3}V}{4\pi \varepsilon_{0}\varepsilon_{r}d}})}{KT}]$$
  where *A* is the area of the heterojunction, A* is the Richardson constant, *d* is the height of the potential barrier at the interface, ∅ *_b_* is the height of the potential barrier at T = 0 K, *ε*_0_ is the dielectric constant of the vacuum, *ε_r_* is the dielectric constant of the semiconductor, *K* is the Boltzmann constant, and *q* is the electronic charge.
Direct tunneling:
(3) $$\ln\left(\frac{I}{V^{2}}\right)\propto \ln\left(\frac{1}{V}\right)-\frac{4\pi d\sqrt{2m\emptyset_{b}}}{h}$$
  where ∅ *_b_* is the height of the potential barrier at T = 0 K, *m* is the effective quality of the charge carrier, *d* is the height of the potential barrier at the interface, and *h* is Planck’s constant.
F-N tunneling:
(4) $$\ln\left(\frac{I}{V^{2}}\right)\propto -\frac{1}{V}(\frac{8\pi d\sqrt{2m\emptyset_{b}^{3}}}{h})$$
  where ∅ *_b_* is the height of the potential barrier at T = 0 K, *d* is the height of the potential barrier at the interface, and *h* is Planck’s constant.

At low bias, carrier transport is governed by direct tunneling, a quantum mechanical effect, resulting in a nonlinear relationship between current and voltage [42,43]. The direct tunneling current is influenced by the thickness and height of the tunneling barrier: thinner barriers and lower heights increase the tunneling probability. At higher bias voltages, carrier injection occurs primarily via F-N tunneling, where the logarithmic relationship between current and voltage exhibits a negative slope [44]. As the applied voltage increases, the electron tunneling probability rises, leading to higher currents at elevated voltages [45]. The appearance of inflection points in the current–voltage curve signals a transition from direct tunneling to F-N tunneling [46].

At high temperatures, when a large number of charge carriers gain sufficient energy to overcome the potential barrier, competition between mechanisms results in a decrease in F-N tunneling carriers, and the inflection point voltage is expected to shift to higher values. For LDH, at room temperature, the 0–1.1 V range is direct tunneling, and when the bias voltage is greater than 1.1 V, it is F-N tunneling. At 100 °C, the 0–3.1 V range is direct tunneling, and when the bias voltage is greater than 3.1 V, it is F-N tunneling. At temperatures above 120 °C, the curve maintains a positive slope, indicating a significant increase in thermal emission. Due to the fact that carriers with higher energy can spontaneously cross the interface barrier under the influence of an electric field, tunneling caused by defects and other mechanisms is significantly reduced.

In contrast, for HDH, the transition from direct tunneling to F-N tunneling occurs at all temperatures. At room temperature, −0.81–0 V is direct tunneling, and when the voltage is less than −0.81 V, it is F-N tunneling. Direct tunneling occurs at −1.29–0 V at 100 °C, while F-N tunneling occurs when the voltage is below −1.29 V. Direct tunneling occurs at −3.6–0 V at 120 °C, while F-N tunneling occurs when the voltage is below −3.6 V. At 140 °C, there is strong competition between the direct tunnel and the F-N tunnel. Direct tunneling occurs at −4.2–0 V at 160 °C, while F-N tunneling occurs when the voltage is below −4.2 V. Direct tunneling occurs at −4.5–0 V at 180 °C, while F-N tunneling occurs when the voltage is below −4.5 V. This behavior may be attributed to higher carrier concentration and narrower electric field caused by heavy doping.

**Figure 9:**
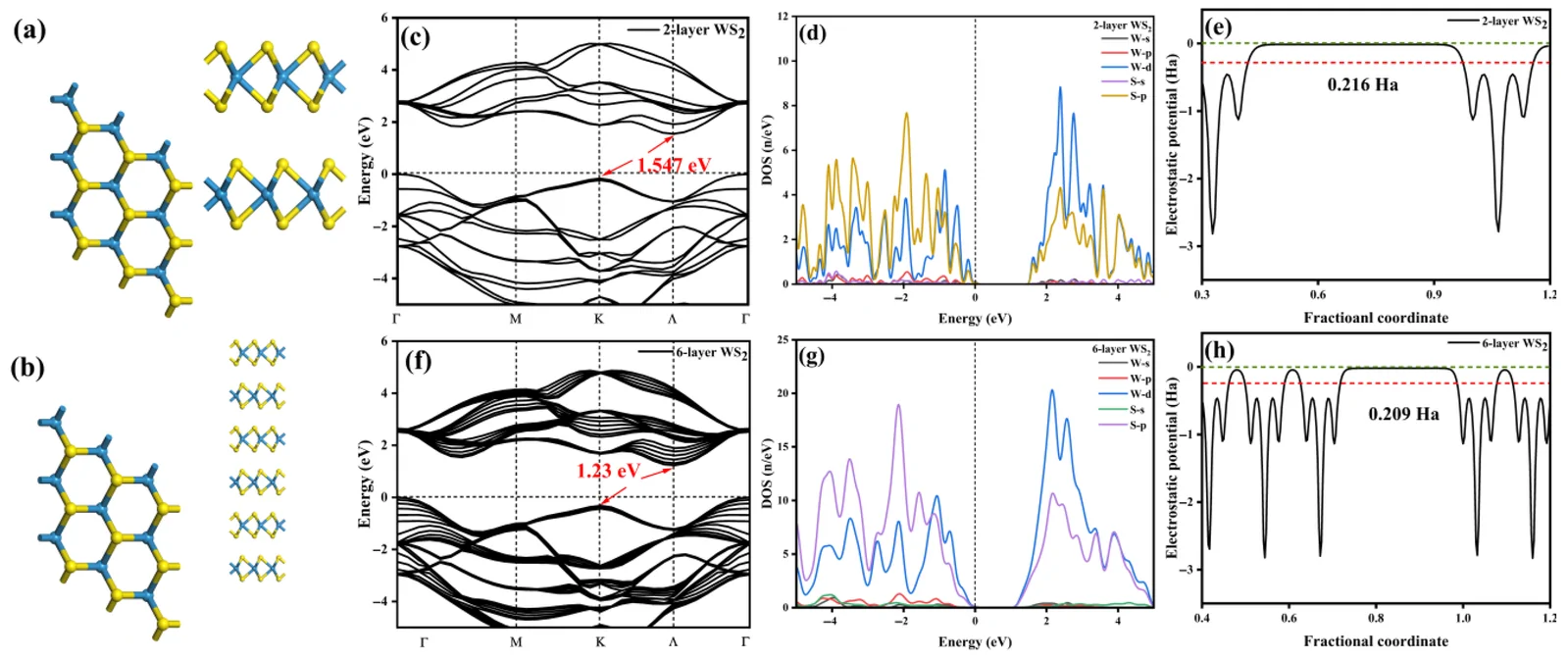
(**a**) Structure of 2-layer WS_2_. (**b**) Structure of 6-layer WS_2_. (**c**) Band structure of 2-layer WS_2_. (**d**) Density of states of 2-layer WS_2_. (**e**) Work function of 2-layer WS_2_. The green line is the vacuum level, and the red line is the Fermi level. (**f**) Band structure of 6-layer WS_2_. (**g**) Density of states of 6-layer WS_2_. (**h**) Work function of 6-layer WS_2_. The green line is the vacuum level, and the red line is the Fermi level.

To investigate the underlying causes of the opposite direction of conductivity, the focus is placed on examining the influence of diamond surface terminations. Initially, convergence tests were conducted on WS_2_ layers. After structural relaxation, the W-S bond length was found to be 2.45 Å, with lattice constants a = b = 3.1842 Å, as shown in Figure 9a,b. Figure 9c–h present the energy bands, density of states, and work function of 2-layer and 6-layer WS_2_. Both 2-layer and 6-layer WS_2_ are indirect bandgap materials, with the highest point of the valence band located at the Γ point and the lowest point of the conduction band located at the Λ point. No new band formation was observed, with a bandgap difference of approximately 0.317 eV between the two. The conduction band is primarily formed by the hybridization of the d orbitals of W and the p orbitals of S [47].

In addition, we measured the electronic states involved in the bandgap transition process in our absorption spectroscopy experiment. Furthermore, the work function difference between the two layers and the six-layer WS_2_ is only 0.007 Ha, and the bandgap width difference is 0.317 eV. It is worth mentioning that the calculation of the bandgap width of WS_2_ can only achieve numerical convergence in four or more layers [48]. However, the numerical value of 0.317 eV is much smaller than that of diamond and WS_2_. Therefore, according to the calculated bandgap width, we established the schematic diagram of the energy band structure as shown in Figure 12 and made a qualitative analysis of the heterojunction. Additionally, here, we mainly focus on the work function, so the bandgap has relatively little influence on the subsequent analysis. Therefore, in order to balance the computational efficiency, the follow-up research will focus on the two-layer WS_2_ model.

**Figure 10:**
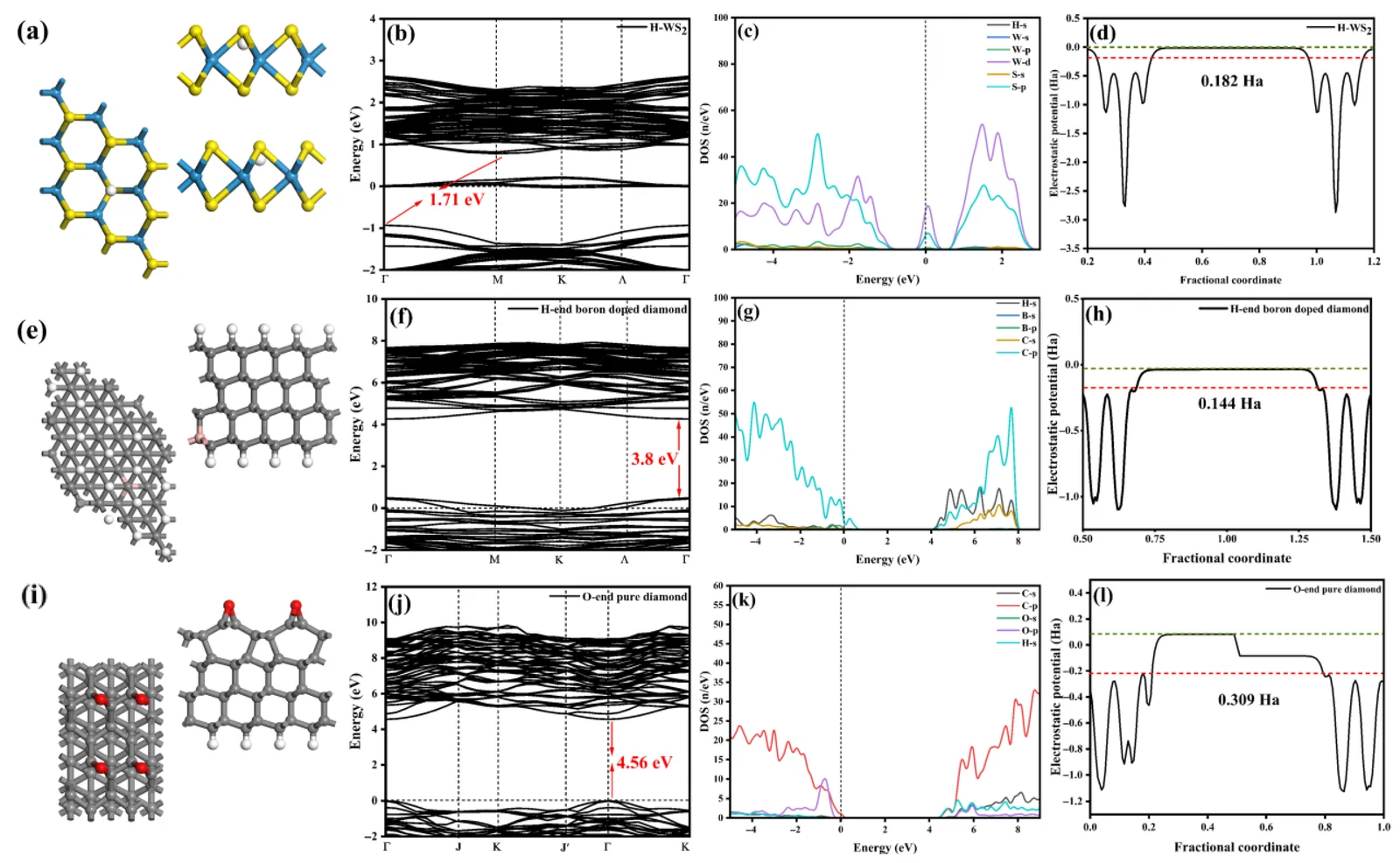
(**a**) Structure of H-doped WS_2_. (**b**) Band structure of H-doped WS_2_. (**c**) Density of states of H-doped WS_2_. (**d**) Work function of H-doped WS_2_. The green line is the vacuum level, and the red line is the Fermi level. (**e**) Structure of H-end boron-doped diamond. (**f**) Band structure of H-end boron-doped diamond. (**g**) Density of states of H-end boron-doped diamond. (**h**) Work function of H-end boron-doped diamond. The green line is the vacuum level, and the red line is the Fermi level. (**i**) Structure of O-end pure diamond. (**j**) Band structure of O-end pure diamond. (**k**) Density of states of O-end pure diamond. (**l**) Work function of O-end pure diamond. The green line is the vacuum level, and the red line is the Fermi level.

The origin of the weak n-type conductivity behavior in WS_2_ remains uncertain, but a widely accepted hypothesis attributes it to unintentional H doping [49]. In this context, a structural model has been proposed where hydrogen atoms fill S vacancies [50]. As shown in Figure 10a–d, the concentration of sulfur vacancies is approximately 3.7%. After hydrogen replaces sulfur, WS_2_ retains its indirect bandgap characteristics. The bottom of the conduction band shifts from the Λ point to the M point, while the top of the valence band remains unchanged. Consequently, the bandgap expands to 1.71 eV.

Simultaneously, a donor level, which is incompletely occupied, is introduced into the bandgap. This donor level is primarily contributed to by the p orbitals of sulfur and the d orbitals of tungsten, with its energy located near the conduction band. This positioning makes it susceptible to thermal excitation. Additionally, hydrogen doping results in a decrease in the work function to 0.182 Ha.

Diamond films synthesized in a hydrogen-rich environment exhibit H-termination characteristics, where each carbon atom is bonded to a hydrogen atom, preserving Sp^3^ hybridization. However, under high-temperature air annealing or ultraviolet irradiation, the films undergo oxidation reactions, and ozone (O_3_) can further accelerate this oxidation process, leading to the formation of oxygen terminations [51]. The formation of oxygen terminations is associated with surface recombination phenomena, akin to the (2 × 1) recombination observed in silicon, with the most stable configuration being the Pandey chain [52]. Oxygen and carbon can form double bonds with 1 M coverage or ether bonds with 0.5 M coverage [53,54]. Among these, the oxygen bridge (C-O-C) has the lowest surface energy, making it the most stable configuration.

Considering that the doping concentration of HBDD is estimated to be 0.5%, we have simulated two extreme cases, 1.5% B-doped H-terminated diamond and pure O-terminated diamond, as shown in Figure 10e,i. The internal six layers of carbon in the model represent the bulk phase, with only the top two layers of carbon allowed to fully relax. The bottom of the O-terminated diamond model is passivated with hydrogen atoms to maintain Sp^3^ hybridization.

As shown in Figure 10e–h, the Fermi level of H-terminated boron-doped diamond enters the valence band, with the valence band top mainly contributed by the p orbitals of C and the conduction band bottom contributed by the s orbitals of H, exhibiting a p-type direct bandgap semiconductor with a bandgap width of 3.8 eV and a surface work function of 0.144 Ha [55]. As shown in Figure 10i–l, the valence band top of O-terminated diamond is mainly contributed by the P orbitals of C and O, while the conduction band bottom comes from the s orbitals of the bottom H and the p orbitals of C, with a bandgap width of 4.56 eV and a work function of approximately 0.309 Ha [56].

**Figure 11:**
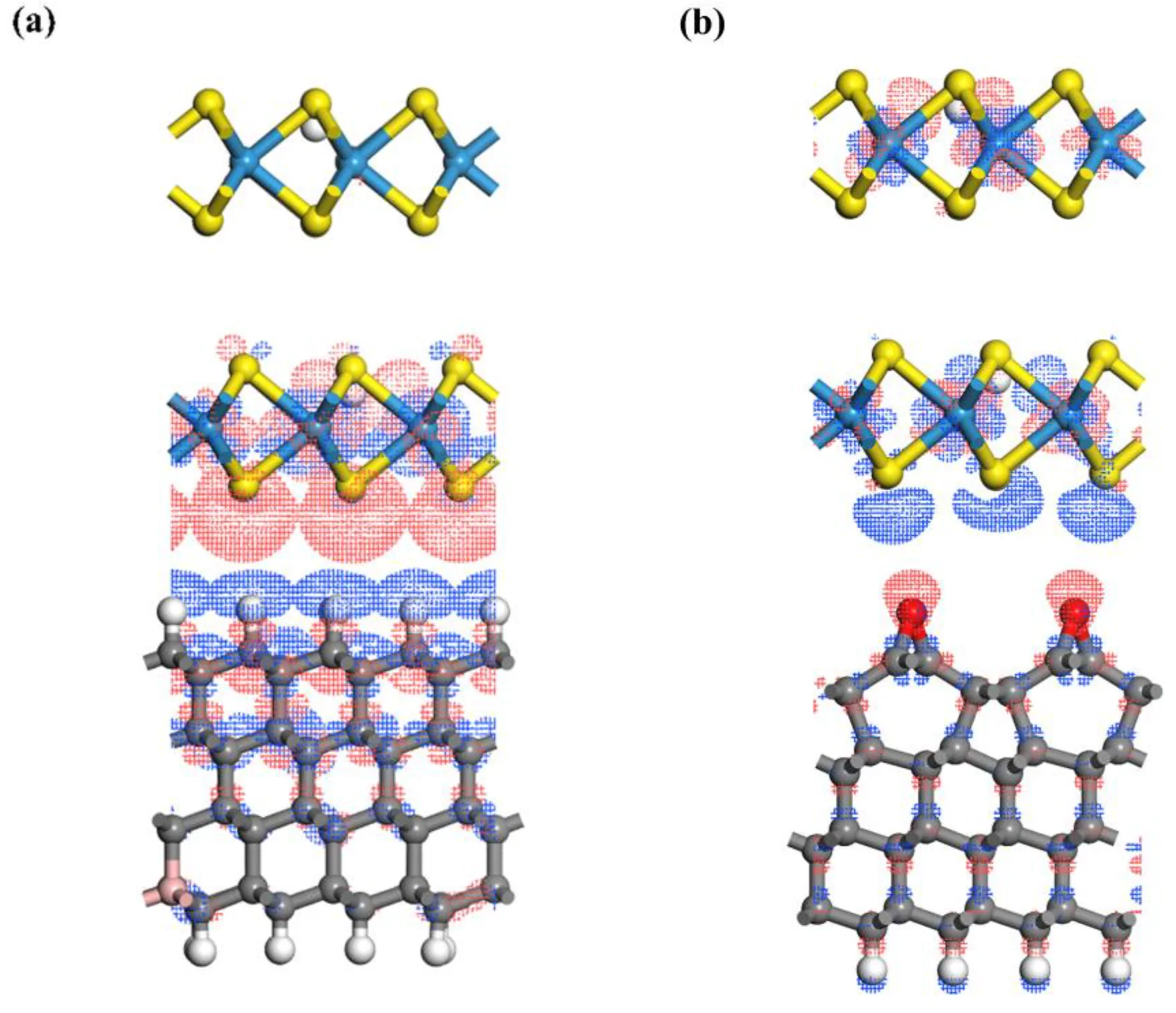
(**a**) Differential charge density of H-terminated boron-doped diamond and H-doped WS_2_ heterojunction. (**b**) Differential charge density of O-terminated pure diamond and H-doped WS_2_ heterojunction.

The work functions of both pure and defective WS_2_ are bounded between those of H-terminated and O-terminated diamond. As a result, when the two materials come into contact, the flow of electrons during Fermi level alignment occurs in opposite directions, leading to distinct built-in electric fields [24]. To visually illustrate this phenomenon, differential charge density calculations were performed for both heterojunction structures. As shown in Figure 11, in the H-terminated heterojunction, electrons accumulate on the WS_2_ side at the interface, while they are depleted on the H-terminated diamond side. In contrast, in the O-terminated heterojunction, electrons are depleted on the WS_2_ side at the interface, while they accumulate on the O-terminated diamond side.

These results indicate that H-termination and O-termination lead to completely opposite rectification characteristics. Furthermore, the electrical experiment shown in Figure 5 fully supports this conclusion. Given that we did not perform any specialized surface treatment, it is likely that LDH formed an O-dominated surface termination under exposure to air and sunlight.

Based on the Anderson model, schematic diagrams of the band structures of LDH and HDH at different temperatures were established. DFT calculations estimated the electron affinity of WS_2_ to be approximately 4.15 eV, that of O-terminated LBDD to be approximately 3.85 eV, and that of H-terminated HBDD to be approximately −0.35 eV. Therefore, before forming the heterojunction, the conduction band offset of LDH is 0.3 eV, and the valence band offset is 2.55 eV. In HDH, the conduction band offset is 4.5 eV, and the valence band offset is 2.41 eV.

In LDH, an initially large valence band offset exists, and due to the lightly doped nature of LDH, it forms an equilibrium band structure as shown in Figure 12a–c. At room temperature, F-N tunneling from the LBDD valence band to the WS_2_ valence band dominates. The difference in carrier concentrations between the materials leads to a higher rectification ratio. When the temperature exceeds 100 °C, a significant number of carriers generated by thermal excitation are able to pass through the interface, thereby enhancing the thermionic emission current [57]. Additionally, the Fermi level shifts toward the center of the bandgap, causing a sharp increase in the concentration of minority carriers [11]. The increase in reverse current becomes more pronounced, ultimately resulting in a decrease in the rectification ratio. As the temperature continues to rise, the excess current and drift current further increase, causing the rectification ratio to stabilize.

In HDH (as shown in Figure 12d–i), the conduction band of WS_2_ and the valence band of HBDD exhibit a band offset of 0.7 eV, with HBDD forming a degenerate semiconductor due to heavy doping, causing the Fermi level to shift from the bandgap region into the valence band [58]. As a result, HDH ultimately establishes a staggered band alignment, as shown in Figure 12d–f. Due to the large band offsets in both the conduction and valence bands, the tunneling current becomes dominant. At room temperature, the number of minority carriers is small, and there may be potential transport paths from the conduction band of WS_2_ to the valence band of diamond, leading to a small reverse rectification [59].

As the temperature increases, the number of minority carriers changes dramatically, and the strong F-N tunneling characteristics further contribute to a significant increase in the rectification ratio. When the temperature exceeds 160 °C, carriers gain sufficient thermal energy to overcome the interface barrier. This leads to an increase in overcurrent and diffusion current, which results in a decrease in the rectification ratio. At 120 °C, when a bias voltage is applied, the conduction band of WS_2_ and the defect energy levels shift upward, enabling carriers to tunnel from the valence band of diamond to the defect energy level band of WS_2_ [60]. This suggests that tunneling current can flow through the interband channel, with peak current occurring when the energy bands align, as shown in Figure 12g–i. If a forward bias voltage is further applied, it bypasses the energy bands, resulting in a decrease in tunneling current. This leads to a negative impedance effect, which approaches the valley current. As the bias voltage continues to increase, the energy bands no longer align, and conventional diffusion current, tunneling current, and excess current begin to jointly dominate the diode current.

## 4. Conclusions

In summary, WS_2_ nanosheet films were assembled onto LBDD and HBDD substrates using electrophoretic deposition, and the electrical properties of the heterojunctions were evaluated at high temperatures ranging from room temperature to 180 °C. The heterojunction with light doping exhibited forward rectification characteristics, with the highest rectification ratio observed at room temperature. Under heavy doping, the heterojunction transitioned to unconventional reverse rectification behavior and demonstrated a negative impedance effect. Both types of heterojunctions displayed similar trends with temperature variation. As the number of thermally excited carriers increased, the current exhibited an upward trend. Compared to LDH, HDH exhibited a larger bias current. The *I-V* characteristics of the heterojunctions are influenced by the shift of the Fermi level at high temperatures and the mechanism of composite carrier injection.

DFT calculations reveal that oxygen capping dictates the forward rectification characteristics of LDH, while hydrogen capping determines the reverse rectification characteristics of HDH. Additionally, sulfur vacancy defects contribute to the emergence of negative differential resistance. HDH exhibits more stable electrical properties at high temperatures, making it suitable for operation in high-temperature environments. The fabricated heterojunctions offer a promising high-performance design for optoelectronic nanodevices intended for use in demanding environments, such as those requiring nanoscale, high-frequency, and high-voltage conditions.

## Figures and Tables

**Table 1:** *I-V* behavior of a lightly doped heterojunction at variable temperature.

Temperature (°C)	RT	100	120	140	160	180
Rectification ratio	9.1	1.1	1.3	1.2	1.3	1.4
Turn on voltage (V)	0.9	0.3	0.2	0.2	0.1	0.1
Ideality factor	16.4	13.8	13.9	14.0	12.7	12.8

**Table 2:** *I-V* behavior of a heavily doped heterojunction at variable temperature.

Temperature (°C)	RT	100	120	140	160	180
Rectification ratio	0.81	0.27	0.99	0.17	0.42	0.44
Turn on voltage (V)	1.3	1.0	0.2	0.5	0.1	0.1
Ideality factor	13.5	11.8	13.5	7.1	12.5	17.7

## Data Availability

The data that support the findings of this study are available from the corresponding author upon reasonable request.
