# Correction: Li et al. High-Temperature Electrical Transport Behavior of p-Doped Boron Diamond Film/n-WS_2_ Nanosheet Heterojunction. *Nanomaterials* 2025, *15*, 1900

**DOI:** 10.3390/nano16171108

**Published:** 2026-09-03

**Authors:** Changxing Li, Dandan Sang, Yarong Shi, Shunhao Ge, Lena Du, Qinglin Wang

**Affiliations:** 1 School of Physics Science and Information Technology, Key Laboratory of Quantum Materials Under Extreme Conditions in Shandong Province, Liaocheng University, Liaocheng 252000, China; 2 Department of Physics, Capital Normal University, Beijing 100048, China

In the original publication [1], there were several errors due to the manuscript not being updated in a timely manner during proofreading. After careful self-examination and review, the authors would like to update the manuscript to correct the following errors.

In the second paragraph of Section 2 “Materials and Method”, the corrections are as follows:

Thin films of lightly doped boron diamond (LBDD) and heavily doped boron diamond (DBDD) were fabricated using hot wire chemical vapor deposition (HWCVD). The heat source was provided by a metal wire, with trimethyl borate and methane serving as the boron and carbon sources, respectively. The working pressure was maintained at 5–8 kPa, and the deposition process lasted for 4.5 h. During this time, the working temperature was kept at 700–900 °C.

In the 18th paragraph of section “Results and Discussion”, the peak voltage of differential negative resistance should be 6.3 V. The corrections are as follows:

At 120 °C, a differential negative differential resistance (NDR) phenomenon is observed, with a peak voltage of 6.3 V, a valley voltage of 8.0 V, and a peak-to-valley ratio of 2.4.

In the 28th paragraph of Section 3 “Results and Discussion”, the position of the top of the valence band of WS_2_ did not move after the defect was introduced. A vacuum layer was applied in the direction C, so the description of the lattice constant in the C direction is inappropriate. The corrections are as follows:

After structural relaxation, the W-S bond length was found to be 2.45 Å, with lattice constants a = b = 3.1842 Å, as shown in Figure 9a,b.

In the 30th paragraph of Section 3 “Results and Discussion”, the sentence “The bottom of the conduction band shifts from the Λ point to the M point, while the top of the valence band moves from the K point to the Γ point” should be changed to “The bottom of the conduction band shifts from the Λ point to the M point, while the top of the valence band remains unchanged.”

The authors state that the scientific conclusions are unaffected. This correction was approved by the Academic Editor. The original publication has also been updated.

## References

[1] Li C., Sang D., Shi Y., Ge S., Du L., Wang Q. (2025). High-Temperature Electrical Transport Behavior of p-Doped Boron Diamond Film/n-WS_2_ Nanosheet Heterojunction. Nanomaterials.

